# Correlation of plasma interleukin-18 concentration and severity of renal involvement and disease activity in systemic lupus erythematosus

**DOI:** 10.15171/jnp.2016.05

**Published:** 2015-11-08

**Authors:** Mohammad Reza Jafari-Nakhjavani, Sima Abedi-Azar, Babak Nejati

**Affiliations:** ^1^Connective Tissue Diseases Research Center, Tabriz University of Medical Sciences, Tabriz, Iran; ^2^Kidney Research Center, Tabriz University of Medical Sciences, Tabriz, Iran

**Keywords:** Systemic lupus erythematosus, Lupus nephritis, Interleukin-18

## Abstract

*Background:* Systemic lupus erythematosus (SLE) is a systemic autoimmune disease characterized by activation of T and polyclonal B lymphocytes. IL-18 was originally identified as a factor which enhances IFN-γ production and is a potent inducer of the inflammatory mediators by T cells, causing severe inflammatory disorders in SLE.

*Objectives:* This study aimed to evaluate the association of plasma interlukine-18 (IL-18) concentration and severity of lupus nephritis (LN) and disease activity in SLE patients.

*Patients and Methods:* In this cross-sectional study, 113 patients with SLE and 50 healthy individuals were examined. Serum level of IL-18 was measured. The severity and activity of the disease was determined by Systemic Lupus Erythematosus Disease Activity Index (SLEDAI) score. The severity of kidney involvement was studied by renal biopsy, serum creatinine and 24 hours urine protein level.

*Results:* The mean level of serum IL-18 was significantly higher in the patients than controls (577.67 ± 649.95 versus 60.48 ± 19.53 pg/ml; *P* < 0.001). In SLE patients with active disease level of serum IL-18 was significantly higher than chronic disease (622.77 ± 716.54 versus 182 ± 184.37 pg/ml; *P* < 0.001). The serum level of IL-18 was significantly higher in stage IV (*P* < 0.001) and V (*P* < 0.001) of patients with LN, than other stages.

*Conclusions:* The current study showed that the serum IL-18 is significantly higher in the patients than controls and it significantly correlated with sever renal involvement and disease activity in SLE patients.

**Implication for health policy/practice/research/medical education:**

In a study on 113 patients with systemic lupus erythematosus and 50 healthy individuals, we found, serum IL-18 is significantly higher in the patients than controls and it is significantly correlated with severity of renal involvement and disease activity in SLE patients.

## 1. Background

Systemic lupus erythematosus (SLE) is an autoimmune disease, which is characterized by the activity of the polyclonal T and B-lymphocytes, production of autoantibodies and formation of immune complexes (1). It has been indicated that a complex network of cytokines control the growth, distinction and induction performance of the B and T cells. A complex network of cytokines is involved in the pathogenesis and activation of SLE, may be a lack of balance in the Th1 and Th2 immune responses and cytokines which leads to activation of B lymphocyte cells is the possible mechanism for the triggering or activation or progression of SLE.

Various studies had shown an unusual level of Th-1 and Th-2 cytokines contributes to the pathogenesis of autoimmune diseases such as SLE. However, data regarding decreasing or increasing of blood level of Th1 cytokines (such as IL-2, IL-12, INF-γ, and TNF-a) and regulatory Th2 cytokines (such as IL-4 and IL-10) are completely conflicting (2-5). Therefore, the lack of consistency, shows the complexity of the cytokine responses of Th lymphocyte cells in SLE patients which requires more investigations. IL-18 is part of the IL-1 family and is a new Th-1 cytokine which is produces by different kinds of cells like Kupffer cells, keratinocytes, intestinal epithelial cells, osteoblasts, adrenal cortex cells and active macrophages. This cytokine plays a major role in the Th1 response to toxic shocks and has common functional similarities with IL-12 (6). IL-12 can stimulate the production of IL-18 and have synergies with IL-18 in the activation of NK cells and cytotoxic T lymphocyte (CTL) cells. It has been reported that IL-18 directly can affect NK and T lymphocyte cells. Therefore, it can lead to the Th2 immune response in the absence of IL-12 (7). The basic functions of IL-18 includes; stimulating the production of INF-γ from T and NK cells, increasing the regulation of the Th-1, IL-2, INF-γ and granulocyte-colony stimulating factor; stimulating the proliferation of activated T cells (6), and also increasing the incidence of Fas ligands on NK and CTL cells (8). The level of Il-18 increases in various human diseases such as rheumatoid arthritis, primary biliary cirrhosis (PBC), autoimmune hepatitis, Crohn disease, lymphohistiocytosis, leukemia, encephalomyelitis and SLE patients (8-11). It is also shown that the variations in level of IL-18 is associated with activity of the disease and severity of renal involvement (8).

## 2. Objectives

Considering the importance of this entity, and to understand the applied role of IL-18 in clinical assays and follow-up of patients, we aimed to determine the possible relationship between IL-18 and renal involvement and disease activity in SLE patients.

## 3. Patients and Methods

In a cross-sectional descriptive-analytical study, which was carried out in the internal disease department of Tabriz University of Medical Sciences on patients with SLE. The level of IL-18 in SLE patients and its relationship with activity of SLE and severity of renal involvement were examined.

In this research, 113 patients with SLE and 50 healthy individuals was tested.

Ninety-eight patients had renal involvement. After obtaining the informed consent of the participants the required information was collected and the Systemic Lupus Erythematosus Disease Activity Index (SLEDAI) score (12-14) was determined using the appropriate form for each participant.

The blood samples of patients and control group were centrifuged and the serum component was separated and stored in a refrigerator at a temperature of -70ºC for the measurement of cytokine levels.

The serum level of IL-18 in patients and control group was measured using the ELISA method by England Bender-Med System Company made ELISA kit. The age, gender, and serum levels of erythrocyte sedimentation rat (ESR), C-reactive protein (CRP), and creatinine levels was measured using standard kits. Urine analysis was conducted for all patients. CBC was also assessed. Accordingly, the anti-nuclear antibody (ANA) status, anti-ds DNA, and levels of C3, and C4 was assessed too. In SLE patients, the activity of the disease and SLEDAI score was calculated.

In cases of renal involvement, renal biopsy was conducted. In renal biopsy we determined lupus nephritis (LN) stages according to World Health Organization (WHO) classification (15). We graded renal biopsies for features of activity (potentially reversible lesions) and chronicity (irreversible lesions). According to National Institutes of Health (NIH) system, for an activity index, the biopsy is graded on a scale of 0 to 3+ for each of six histologic features including endocapillary proliferation, glomerular leukocyte infiltration, wire loop deposits, fibrinoid necrosis and karyorrhexis, cellular crescents and interstitial inflammation. Extracapillary proliferation (crescent), and fibrinoid necrosis are assigned double weight. The sum of the individual components yields a total histologic activity index (AI) score of from 0 to 24. In this regard we divided them to 4 groups; (group 1 AI: 0-6, group 2 AI: 6-12, group 3 AI: 12-18 and group 4 AI: 18-20).

Central nervous system (CNS) involvement, treatment type, and dosage of prednisolone was also noted.

Inclusion criteria was the diagnosis of SLE based on new American college of rheumatology (ACR) criteria (16). Patients with overlap or other inflammatory disease were excluded from the study.

### 3.1. Ethical issues

The research followed the tenets of the Declaration of Helsinki; 2) informed consent was obtained, and they were free to leave the study at any time and; 3) research was approved by the ethical committee of Tabriz University of Medical Sciences. Throughout the whole study project no financial or physical damage was caused to the patients. Moreover, no additional expenses was also imposed on the patients for examination of IL-18 levels. All of the patient information also remained confidential.

### 3.2. Statistical analysis

The obtained information is expressed in terms of mean SD and frequency (%).

The qualitative (categorical) variables were compared and analyzed using the contingency tables and the chi-square test or Fisher exact test. The quantitative variables were compared using the t test and one-way analysis of variance (ANOVA) or Mann-Whitney U tests depending on the distribution in SPSS. The level of significance was *P *< 0.05 for all compared variables. Data were analyzed using SPSS version 22 (SPSS Inc., Chicago, USA).

## 4. Results

**Figure 1:**
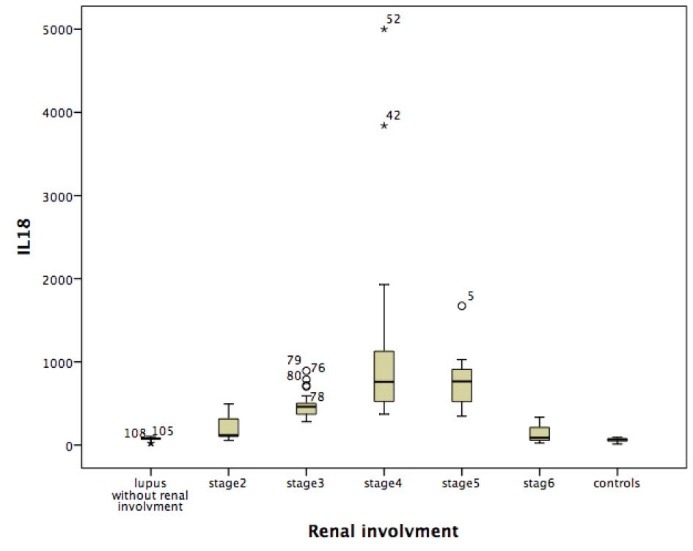
IL-18 level in patients with different stages of renal involvement and controls.

Of patients with SLE, 103 were female and 10 were male. In the control group, 43 were female and 7 were male (*P *= 0.218). The mean age of patients with SLE and patients in the control group was 30.74 ± 10.49 and 29.48 ± 7.20 years, respectively (*P *= 0.106). The mean serum level of IL-18 in patients and control group were 577.67 ± 649.95 and 60.48 ± 19.53, respectively, which in patients was significantly higher than the healthy individuals (*P *< 0.001). Moreover 4.4% of patients had chronic disease, 13.3% of patients had a mild disease activity (SLEDAI score <4), while, 82.3% of patients had relatively high disease activity (SLEDAI score >4). The mean serum levels of IL-18 in patients with chronic disease, patients with slight activity and patients with active disease were 109.17 104.63, 182.00 ± 184.37 and 622.77 ± 716.54, respectively. Patients with active disease was significantly higher IL-18 than other SLE patients, with chronic or slight activity index (*P *< 0.001). In addition, 99 (87.61%) (F=92; M=7) of patients had renal involvement according to the renal biopsy. Lupus nephropathy of stage II in 19 patients, stage III in 27 cases, stage IV in 37 cases, stage V in 13 patients and finally stage VI was observed in 3 cases. Mean ± SD level of IL-18 in patients with renal involvement was 649.58 ± 663.72 pg/ml and in patients without renal involvement it was 69.21 ± 27.74 pg/ml, the later although is higher than controls but it is not statically significance (*P *< 0.12). The mean serum level of IL-18 in patients with stage IV of LN (1007.86 ± 915.93 pg/ml) was significantly higher in comparison with stage II (192.95 ± 144.64 pg/ml) (*P *< 0.001), stage III (479.67 ± 151.39 pg/ml) (*P *< 0.001), stage V (765.54 ± 357.04 pg/ml) (*P *< 0.001) and stage VI (149.33 ± 163.09 pg/ml) (*P *< 0.001) (Figure 1). Additionally a significant correlation of IL-18 level with stage V of LN was observed (*P *< 0.004). To find the relationship of serum IL-18 and the severity of pathologic findings, we compared 4 groups of activity index (group 1: 0-6, group 2: 6-12, group 3: 12-18 and group 4: 18-20) with serum IL-18 level. The results was summarized in Table 1. In stages III and IV of LN mean level of IL-18 in groups with AI between 12-18, and 18-24 were significantly higher than lower activity index (*P *< 0.01 and *P *< 0.001, respectively) (Table 1). In this study, LN in 87.61% of patients, cardiac involvement (i.e. pericardial effusion), in 9.7% and 28.9% with CNS involvement was found. CNS presentations, consisted of seizure in 12.4%, psychosis in 5.3%, cerebrovascular accident (CVA) in 1.8%, retinal vasculitis in 2.7%, cerebral vasculitis in 1.8%, psychosis 1.4% and finally seizure along with headache in 3.5% of SLE patients was seen. There was not significant relationship between serum IL-18 level and patients’ age (r* *= -0.04, *P *= 0.6), ESR (r* *= 0.1, *P *= 0.3), serum level of C3 (r* *= -0.1, *P *= 0.1) and C4 (r* *= -0.083, *P *= 0.568). A significant direct linear association between serum level of IL-18 and twenty-four urine protein level (r* *= 0.4, *P *= 0.01), and serum creatinine (r* *= 0.3 *P *= 0.01) and also with SLEDAI (r* *= 0.3, *P *< 0.001) was detected. More over the association of IL-18 with serum anti-ds DNA levels in our patients was significantly positive (r* *= 0.7, *P *< 0.001). Of total SLE patients, 22 (19.4%) were anti-ds DNA negative. The mean serum level of IL-18 in anti-ds DNA negative patients (332.78 ± 204.36 pg/ml) was significantly lower than anti-ds DNA positive patients (697.12 ± 846.43 pg/ml; *P *= 0.04).

## 5. Discussion

In this study, the serum level of IL-18 in 113 patients with SLE and 50 healthy participants was tested. The mean serum level of IL-18 in the patient group was significantly higher than the healthy group (577.67 ± 649.95 versus 60.48 ± 19.53 pg/ml; *P *< 0.001). The study by Chu et al (17) which was carried out on 80 patients with SLE and 40 healthy individuals, and the study of Wong et al (18) and Wong et al (19) shows that IL-18 may play a role in the complex pathogenesis of SLE (20,21), results of our study from this point of view comply with the results of similar studies. In this study, we also found the mean serum level of IL-18 in anti-ds DNA negative patients was significantly lower than anti-ds DNA positive patients. The result was similar to Mosaad et al study which was carried out on 32 SLE patients (22). However, in the study of Chan et al on patients with active LN significant relationship between IL-18 and anti-dsDNA was not observed (23,24).

In our study, a significant relationship of serum level of IL-18 and the degree of involvement score (SLEDAI) (ho=0.277, *P *= 0.05) was found. Furthermore, the mean level IL-18 in patients with active SLE was significantly higher than patients with chronic SLE (622.77 ± 716.54 versus 109.17 ± 104.63 pg/ml, *P *= 0.001), such a results have been shown in the studies by Wong et al (20,21,24) and Aringer et al (25). Although in some other studies (e.g. Robak et al) no significant statistical relationship between IL-18 level and severity of the disease was observed (26). Interestingly, in the analysis of the relationship between serum level of IL-18 and SLEDAI score in patients with LN, a positive and significant correlation was observed between the two variables of IL-18 and SLEDAI (r* *= 0.466, *P *= 0.008). The same finding was confirmed by some previous studies (20,27) too.

Further studies are required to determine whether the level of IL-18 is related to the severity of LN in patients with SLE. In our study the relationship of serum level of IL-18 and involvement of different organs (such as kidneys, CNS, heart, and hematologic disorders) was found. In this study, LN in 87.61% of patients, cardiac involvement (i.e. pericardial effusion), in 9.7% and 28.9% with CNS involvement was found. The mean serum level of IL-18 in patients with CNS involvement, cardiac involvement, leukocytopenia or thrombocytopenia was significantly higher when they had kidney involvement than SLE patients without renal involvement, even if it was higher than controls. The similar result was concluded in the study of Calvani et al, which they showed, the serum level of IL-18 increases in the presence of renal involvement while involvement of other organs in SLE patients does not impact a significant effect on the mean serum level of IL-18 (28).

In the literature some studies pointed to similar results like Chu et al (17) which studied a total of 80 SLE patients with and without LN. They found significantly higher level of IL-18 in group with renal involvement (27). Moreover Wong et al in a study on 35 patients with LN and 37 patients without LN found, the mean serum level of IL-18 in the patients with renal involvement was significantly higher than the group of SLE patients without obvious kidney involvement (20).

We studied the relationship between different stages of LN and serum IL-18 level. We found, the highest level of IL-18 in stage IV (1007.86 ± 915.93 pg/ml) which was followed by the stage V (765.54 ± 357.04) of LN. This result was also mentioned in the study of Tucci et al (29). In this study, we indicated a direct relationship between serum level of IL-18 and high activity indexes (AI>18) (*P* > 0.001). Hence, it is possible that a relationship between level of IL-18 and severity of renal involvement in patients with SLE existed. In this regard, comprehensive pathological studies should be carried out at the molecular level that confirms the existence of this relationship. Moreover, in our study, there was not significant difference of IL-18 between male and females (648.89 ± 796.08 versus 323.89 ± 332.08 pg/ml; *P *= 0.2).

**Table 1:** Mean±SD of IL-18 level in patients with different AI

	**Stage of LN**					
	**Stage III (n=27)**				**Stage IV (n=37)**		
	**Proportion of patients**	**IL-18 (pg/ml)**	**P value**	**Proportion of patients**	**IL-18 (pg/ml)**	**P value**
**AI**						
0-6	6	343.67±64.39	0.4	1	407	0.25
6-12	11	457.82±84.30	0.03	9	511.56±70.88	0.43
12-18	7	505.71±33.19	0.01	13	751.93±378.47	0.01
18-24	3	771.04±104.99	0.001	8	1673.31±1259.83	0.001

Abbreviations: AI, Activity index; LN, Lupus nephritis‏.

Finally, it should be concluded that IL-18 is an important factor in the pathophysiology and activity of SLE and also involvement of different organs especially the kidneys.

Considering the complexity of the mechanism for the development of SLE and its complications with pointing to some studies of animal models the disease was induced or intensified through infusion of IL-8 (30) and prevented lupus like disease by vaccination of the mice against IL-18 (31). However, more controlled studies are required on this subject to find the precise role of IL-18 on the aggravation of lupus nephropathy.

## 6. Conclusions

The mean serum level of IL-18 in patients with SLE is significantly higher than healthy individuals and it is significantly correlate with disease activity and severity in SLE patients in presence of renal involvement.

## 7. Limitations of the study

The limitations of our study was small sample size, thus more studies with larger sample size are recommended to better understand the exact role of IL-18 in SLE patients.

## Authors’ contribution

BN and MRJN designed the study, observed accuracy and validity of the study. BN and MRJN collected the data and follow the study. SAA wrote the paper. MRJN edited the final manuscript.

## Conflicts of interest

The authors declared no competing interests.

## Funding/Support

This study was extracted from residential thesis of Babak Nejati in Tabriz University of Medical Sciences.
